# Patterns of Immunohistochemical Expression of P53, BCL2, PTEN, and HER2/neu Tumor Markers in Specific Breast Cancer Lesions

**DOI:** 10.1155/2022/2026284

**Published:** 2022-10-17

**Authors:** Mohamed Ahmed Bealy, Awad Aljeed Abugooda, Ruba Mustafa Elsaid Ahmed, Nuha Abdel Rahman Khalil, Abdelbaset Mohamed Elasbali, Ghorashy Eltayeb Yousif Mohamed, Faris Merghani Eltom, Hussain Ahmed

**Affiliations:** ^1^Department Pathology, College of Medici University of Ha'il-Saudi Arabia, Ha'il, Saudi Arabia; ^2^Department Histopathology and Cytology Faculty of Medical Laboratory Sciences (FMLS) University of Kordofan-Elobeid-Sudan, Elobeid, Sudan; ^3^Department of Histopathology and Cytology FMLS University of Khartoum-Sudan, Khartoum, Sudan; ^4^Clinical Laboratory Science, College of Applied Medical Science- Qurayyat, Jouf University, Qurayyat, Saudi Arabia; ^5^Department of Histology, College of Medicine, Alrayan Colleges, Almadinah Almunawwarah, Saudi Arabia

## Abstract

**Objective:**

This study aimed to associate the expression of P53, BCL2, PTEN, and HER2/neu tumor markers in specific breast cancer lesions.

**Methods:**

This study analyzed the immunohistochemical expression of P53, BCL2, PTEN, and HER2/neu tumor markers for 306 patients who presented with lesions. Tissue blocks and patients' identification data were retrieved from the department of pathology, AL Madinah Almonwarah hospital, Al Madinah, UAE.

**Results:**

Of the 306 patients, 104 had benign lesions and 202 had malignancy (including 194 females and 6 males). Most females were presented with invasive ductal carcinoma (IDC), followed by infiltrating ductal carcinoma, and invasive lobular carcinoma (ILC), representing 70%, 23.2%, and 3.7%, respectively. Positive P53, BCL2, PTEN, and HER2 were identified in 20.8%, 11.9%, 91%, and 18.3%, respectively.

**Conclusion:**

: The expression of P53, BCL2, PTEN, and HER2/neu tumor markers among Saudi patients with breast cancer is relatively similar in many parts of the world.

## 1. Introduction

Breast cancer is a worldwide challenge, and nearly 1,700,000 new cases are detected annually. Deaths are rising in developing countries by around 60%, while in America, the number of newly diagnosed cases every year is about 250 thousand, the highest except for skin cancer, and mortality from breast cancer is declining [1]. In Saudi females, breast carcinoma (CA) is one of the most malignancies, with a frequency rate of 53%. A recent study on cancer deaths among Saudi females reveals that breast cancer is the ninth principal cause of death [2, 3]. Many risk factors are known to contribute to breast cancer, such as old age, alcohol, and hereditary. Many mutant genes have a role in breast cancer, i.e., P53, the most known mutant gene in human cancer, and serve in different cellular signals, including cell cycle regulation, metabolism, formation of blood vesicles, and nucleic acid repair. About 30% of breast tumors are suspected of having a mutation in the P53 gene, and recently there has been an indication that the occurrence, range, and period of these mutations differ according to the genetic subtype of the tumor [4]. Based on gene-expression profiling, breast cancer can be classified into luminal, human epidermal growth factor receptor 2 (HER2)-enriched, normal-like, and basal-like subtypes by particular medical and epidemiological features. In general terms, luminal breast cancer has a better prognosis than nonluminal (HER2 expression positive) and more capability to respond to reproductive treatments [5]. The gene of phosphate and tension (PTEN) is a tumor suppressor lipid phosphate, capable of mutation or deletion in breast cancer tissue. Absence of PTEN is linked with abnormal activation of PK/AKT signaling pathways, which leads to failure of cell cycle control, migration, and increased cell life. Hence, normal functional PTEN is important in preventing cancer and metastatic cancer [6].

The Bcl2 gene mainly regulates mitochondrial membrane permeability and carries an anti-cell death function. This gene stops apoptosis and leads to continued cell division without control, with expanding cells having the same behavior. Thus, Bcl2 is one of the cancer enhancing aspects that can induce the role of another ontogenesis [7]. There was a different pattern of breast cancer. These patterns may appear on a particular occasion, i.e., medullary carcinoma most often appears in triple-negative cancer than in nonluminal one HER2 negative [8].

Hence, the current study aimed to associate the expression of P53, BCL2, PTEN, and HER2/neu tumor markers in specific breast cancer lesions.

## 2. Materials and Methods

In this, we analyzed the immunohistochemical expression of P53, BCL2, PTEN, and HER2/neu tumor markers for 306 patients who presented with breast lesions. Tissue blocks and patients' identification data were retrieved from the department of pathology, AL Madinah Almonwarah hospital, Al Madinah, UAE. All specimens were previously histopathologically diagnosed. Histopathology diagnosis was based on the World Health Organization (WHO) classification of tumors of the breast, 4th edition. This WHO tumor classification of the breast covers not only invasive breast cancers but also precursor lesions, lesions of low malignant potential, benign epithelial proliferations, fibroepithelial, myoepithelial and mesenchymal neoplasms, among others. Its encyclopedic character makes it an essential reference for pathologists, clinicians, and researchers on breast cancers alike [9].

Four sections from each paraffin block were cut 4 *μ*m in thickness and floated into a floating water path of 45c. Each section was placed on a coated slide for immunocytochemistry staining.

### 2.1. Immunohistochemistry Staining

IHC was done for all markers P53, Her2, PTEN, and Bcl2 genes adopting the following procedure: Serial sections on poly-L-lysine-coated slides for immunohistochemistry (IHC), one section on a regular slide for hematoxylin and eosin stain, were prepared from each case.

The immunohistochemical staining was performed as follows: slides were heated overnight at 56°C followed by deparaffinization through graded ethyl alcohols and rehydration. Before immunostaining with antibodies, the tissues were treated with 10 mM sodium citrate buffer at 1001°C for 15 minutes for antigenic retrieval.

The samples were incubated with 0.3% hydrogen peroxide in methanol for 30 minutes to inhibit endogenous peroxidase activity and washed 3 times with phosphate-buffered saline (PBS). After blocking nonspecific sites with horse normal serum diluted in phosphate buffer (PBS), the slides were rinsed with distilled water for 2 × 5 minutes. Primary antibodies were incubated for 8 hours in a humidity chamber using the following dilutions: p53, HER-2/neu, BCL2, and PTEN were performed by applying the avidin-biotin-peroxidase complex method. After 2 × 5 minutes in rinse PBS, the secondary antibody (LSAB2, DAKO) was incubated for 30 minutes in the same chamber. According to the manufacturer's instructions, the primary antibody was detected using the Strepto ABC, LSAB2 system (DAKO) [8]. The slides were counter-stained with hematoxylin, mounted, and analyzed with a light microscope. All slides were performed at the same time and submitted to standard methods. Known positive and negative cases were used as external controls [10].

### 2.2. Inclusion and Exclusion Criteria

All diagnosed blocks with breast lesions and complete reports are available, whereas blocks with other lesions than breast or breast with an incomplete diagnosis are excluded.

### 2.3. Statistical Analysis

Data management was done using the Statistical Package for Social Sciences (SPSS version 16). SPSS was used to analyze and perform the Pearson Chi-square test for statistical significance (*P* value). The 95% confidence level and confidence intervals were used, and *P* < 0.05 was considered statistically significant. Cross-tabulations, frequencies, and variable correlations were also calculated using SPSS.

### 2.4. Ethical Approval

Ethical approval was obtained from the College of the Medicine ethical Committee University of Hail. Approval number: HREC 00122/CM-UOH.04/20.

## 3. Results

**Figure 1:**
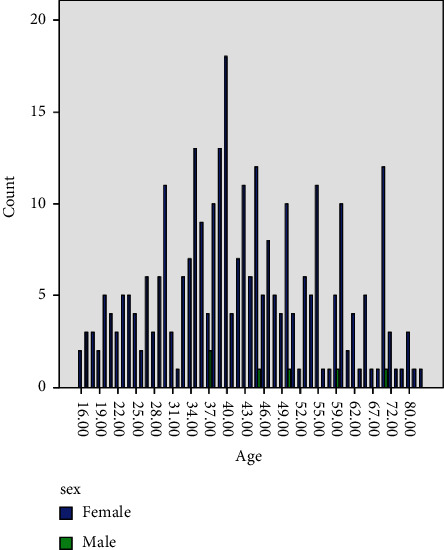
Description of the study population by age and sex.

This study assessed apoptotic tumor suppressor genes (Bcl2, P53, and PTEN) in a series of patients with breast lesions, including 202 with breast cancers, and 104 with benign breast lesions. Of the 202 cancer patients, 6/202(3%) were males, and the remaining 196/202(97%) were females. Cancer patients were aged 25–99 with a mean age of 48, as indicated in Table 1 and Figure 1. All benign cases were identified with negative expression of all three markers.

**Figure 2:**
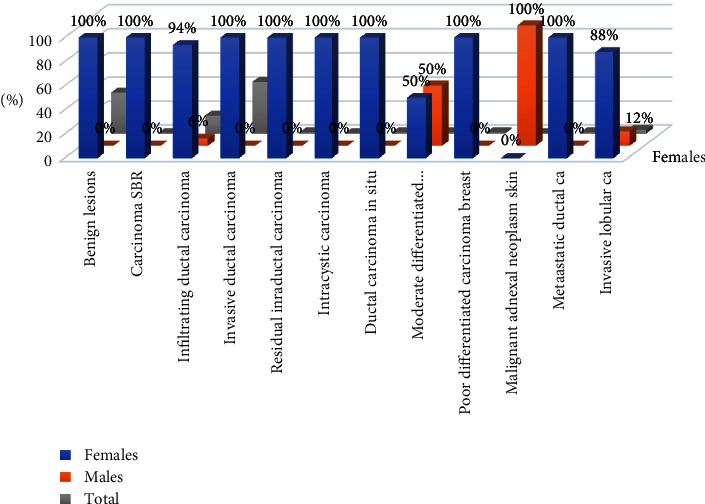
Description of the study population by sex and pathology.

As indicated in Table 2 and Figure 2, out of the 294 female patients, 190/294 (64.6%) were presented with breast cancer, and the remaining 104/294 (35.4%). Most females were presented with invasive ductal carcinoma (DC), followed by infiltrating DC, and invasive lobular carcinoma (LC), representing 133/190 (70%), 44/190 (23.2%), and 7/190 (3.7%), respectively. All males were presented with breast cancer. Most male cancers were seen with infiltrating DC constituting 3/6 (50%), then moderately differentiated adenocarcinoma (MDFC) 1/6 (16.7%), malignant adnexal neoplasm skin 1/6 (16.7%), and ILC 1/6 (16.7%).

**Figure 3:**
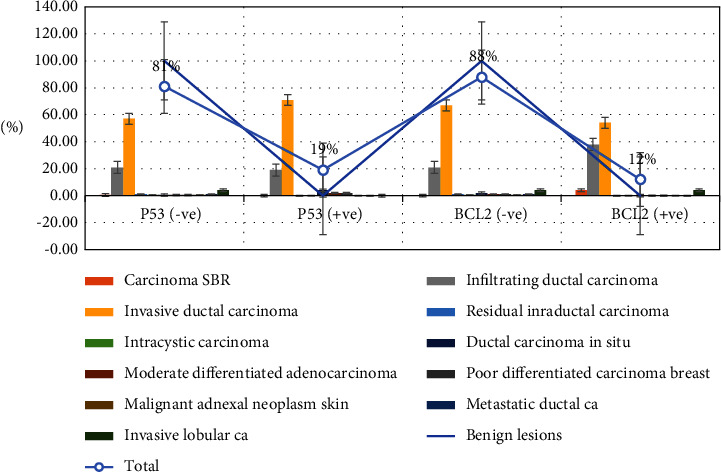
Description of diagnosis by P53 and BCL2 immunohistochemically expression.

Table 3, Figure 3, summarizes the diagnosis distribution by P53 and BCL2 immunohistochemical expression. Positive P53 immunoexpression was identified in 42/202(20.8%) and could not be noticed in 180/202 (89.1%). High P53 expression was seen in IDC, followed by infiltrating ductal carcinoma, constituting 30/42 (71.4%) and 8/42 (19%). BCL2 positive immunoexpression was seen in 24/202 (11.9%) and negative in 178/202 (88.1%). Highly BCL2 expression was seen in IDC followed by Infiltrating ductal carcinoma, representing 13/24 (54.2%) and 9/24 (37.5%), correspondingly.

**Figure 4:**
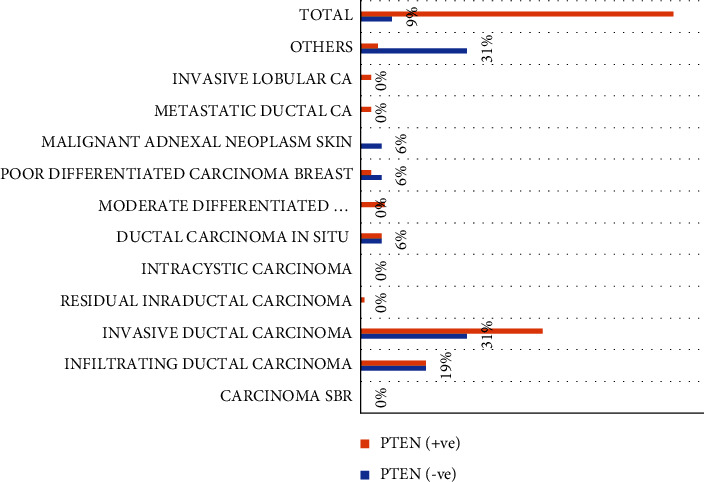
Description of Diagnosis by PTEN immunohistochemically expression.

**Figure 5:**
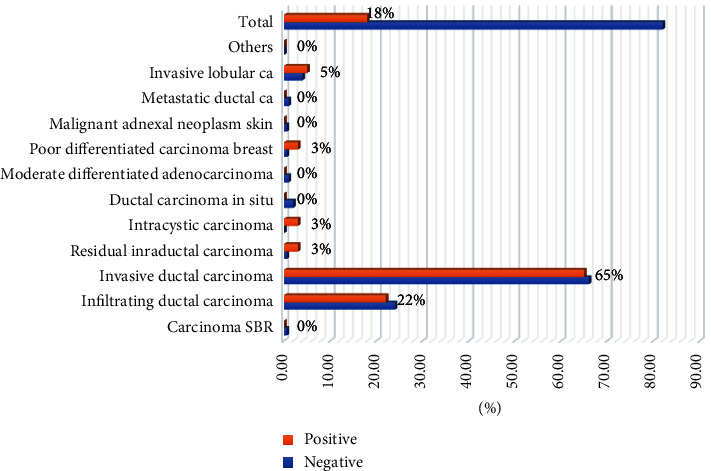
Description of Diagnosis by Her2/neu immunohistochemically expression.

Table 4, Figures 4 and 5 summarizes the distribution of study subjects by PTEN and Her2/neu immunohistochemically expression. Positive immunoexpression of PTEN was detected in 160/176 (91%) and could not be identified in 16/176 (9%). Of the 16 negative cases, 5/16 (31.3%) were IDC, and 3/16 (18.7%) were infiltrating DC.

Positive immunoexpression of Her2/neu was detected in 37/202 (18.3%) and could not be identified in 165/202 (81.7%). Out of the 37 positive cases, 24/37 (65%) were IDC, and 8/37 (21.6%) were infiltrating ductal carcinoma.

**Figure 6:**
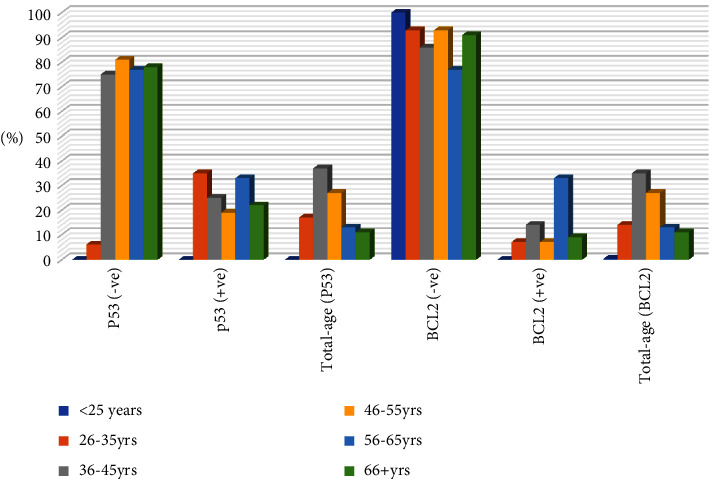
Description of P53 and BCL2 immunohistochemical expression within the entire age group.

**Figure 7:**
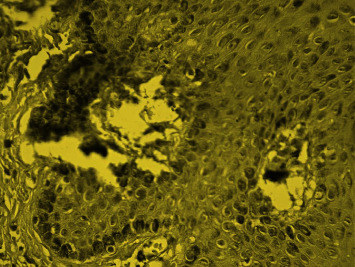
Show positive immunoexpression for Her2 breast ductal carcinoma.

**Figure 8:**
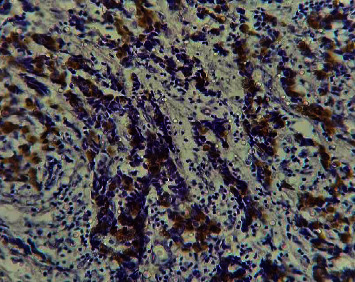
Show positive immunoexpression for P53 breast ductal carcinoma.

As shown in Table 5, Figure 6, P53 positive expression was predominantly noticed in the age range 36–45 years, followed by 26–35 years and 46–55 years, representing 10/42 (23.8%), 9/42 (21.4%), and 8/42 (19%), in that order. Positive BCL2 was mutually observed in the age group 36–45 years, followed by 56–65 years and 46–55 years, representing 10/24 (41.7%), 6/24 (25%), and 4/24 (16.7%) per capita (see Figures 7 and 8).

## 4. Discussion

Tumor markers have a significant role in determining several cancer-related factors. These include predictions of possible etiology, cancer behavior, clinico-pathological indications, treatment, and prognosis. Failure or impairment of apoptosis may significantly elevate the chance of neoplastic propagation. Thus, this study aimed to assess apoptotic tumor suppressor genes (Bcl2, P53, and PTEN) in a series of patients with breast lesions. Benign breast lesions did not show an abnormal expression in all demonstrated markers.

In this study, approximately 21% of the cancer cases showed positive P53 immunoexpression. P53 mutations occur in several cancers, including breast cancer. P53 mutation is usually associated with more aggressive breast cancer characteristics. Still, there is a lack of clinical, pathological, and epidemiological data on breast cancer subtypes and P53 immunoexpression [9]. P53 gene mutations are believed to be associated with 70% to 80% of triple-negative breast cancer cases (TNBC) [11]. In turn, triple-negative breast cancer represents about 12% to 20% of the breast cancer subtypes. TNBC is a subtype of breast cancer that is negative for estrogen (ER), progesterone (PR), and human epidermal growth factor receptor 2 (HER2). The treatment of TNBC is challenging due to its inadequate response to cancer therapies and its highly invasive characteristics [12, 13].

Most positive cases in the present study were seen among invasive ductal carcinoma (71.4%) and infiltrating ductal carcinoma (19%). However, some studies have reported a broad range of P53 expression in invasive breast carcinoma, from 15% to 50% [14, 15], but all were much lower than our findings in this, which might be attributed to the large number of patients with invasive cancer carcinoma in this study.

Positive BCL2 immunoexpression was detected in 11.9% of the patients in this study. BCL2 genes encode proteins responsible for regulating apoptosis (apoptosis regulator) by controlling mitochondrial outer membrane permeability, which acts as an antioncogenic [16]. Some reports have shown that females with BCL2 mutation have a three-times or higher risk of developing breast cancer than others [17]. BCL2 raises the metabolic strength of MCF7 breast cancer cells, which was correlated with heightened mitochondrial NAD (P) H and ATP concentrations. The primary role of BCL2 overexpression in tumor cells is to expand their resistance to metabolic stress in the tumor microenvironment, independent of cell death signaling [16]. Bcl-2 has an anti-apoptotic protagonist, causing unfortunate clinical consequences or resistance to therapy in most tumor types expressing Bcl-2. However, in breast cancer, Bcl-2 expression has been described as a promising prognostic factor. The positive correlation of Bcl-2 with estrogen receptor (ER)/progesterone receptor (PR) status, and endocrine therapy regularly offered for hormone receptor-positive tumors, may confuse the independent pathobiological responsibility of Bcl-2 [18]. On the other hand, Bcl2 might provoke cellular metastasis in breast cancer through the epithelial to mesenchymal transition [19].

Loss of immunoexpression of PTEN was identified in about 8% of breast cancer patients in the current series. In breast cancer, loss of PTEN is usually linked to tumorigenesis, tumor progression, and resistance to therapeutic regimes. A previous study has shown similar PTEN loss of expression findings, observed in 7.4% of breast cancer patients [20]. Some studies have reported that PTEN gene mutations occurr in 5% to 10%, which is in line with our findings in the present study [21, 22]. PTEN immunoexpression loss usually correlates with more significant tumor sizes, multiple lymph node metastases, and an aggressive triple-negative phenotype [23].

Absence of PTEN immunoexpression was found to correlate with ER-negative (*P*=0.021), PR-negative (*P*=0.024), and triple-negative (*P*=0.0024) breast ductal cancers. PTEN inhibits PI3 K, causing reduced downstream effector stimulation and mammalian rapamycin target (mTOR). The absence of PTEN results in cell proliferation through the activation of mTOR. Targeted therapy with mTOR inhibitors may be beneficial in treating triple-negative breast cancer [24].

The present study found about 18.3% of patients with Her2 positive immunoexpression. In most instances, HER2 is a part of triple-negative breast cancer, representing about 24% of the newly diagnosed breast cancers [25].

HER2 is a familiar negative prognostic component in breast cancer and a target of the monoclonal antibody trastuzumab and other anti-HER2 combinations [26]. Triple-negative breast cancer usually represents superior heterogeneity, elevated levels of metastasis, poor prognosis, and absence of therapeutic targets [27].

However, breast cancer is heterogeneous, and its growth is strongly connected to the inherent molecular monitoring network. The key dysregulated networks are extensively developed in critical breast cancer-related pathways and driver genes, strongly associated with drug targets, and significant disparities in survival analysis. Furthermore, the key dysregulated genes might act as likely driver genes, drug targets, and prognostic signs for every single breast cancer subtype [28].

Regarding the epidemiology of these markers in UAE, there is a lack of literature, and our findings in the present study may be helpful signs for future breast cancer management and better design of guidelines and further study settings.

In conclusion, the expression of P53, BCL2, PTEN, and HER2/neu tumor markers among Saudi patients with breast cancer is relatively similar to many parts of the world. There is a lack of distinct literature regarding the epidemiology of these markers and their inclusion in the determination of patient management. Further studies are necessary for better patient treatment and overall management in UAE.

## Figures and Tables

**Table 1:** Distribution of the study population by age and sex.

Variable	Females	Males	Total
<25 years	36	0	36
26–35	58	0	58
36–45	94	3	97
46–55	59	1	60
56–65	29	1	30
66+	24	1	25
Total	300	6	306

**Table 2:** Distribution of the study population by sex and pathology.

Lesion type	Females	Males	Total
Benign lesions	104	0	104
Carcinoma SBR	1	0	1
Infiltrating DC	44	3	47
Invasive DC	133	0	133
Residual intraductal (RIDC)	2	0	2
Intracystic CA	1	0	1
Ductal carcinoma in situ	3	0	3
MDFC	1	1	2
Poorly differentiated carcinoma (PDFC)	2	0	2
Malignant adnexal neoplasm skin (MANS)	0	1	1
Metastatic DA	2	0	2
Invasive LC	7	1	8
Total	300	6	306

**Table 3:** Distribution of diagnosis by P53 and BCL2 immunohistochemical expression.

Lesion type	P53		Total	BCL2		Total
	−Ve	+ve		−Ve	+ve	
Benign lesions	20	0	20	0	0	0
Carcinoma SBR	1	0	1	0	1	1
Infiltrating DC	39	8	47	38	9	47
Invasive DC	103	30	133	120	13	133
RICA	2	0	2	2	0	2
Intracystic carcinoma	1	0	1	1	0	1
DC in situ	1	2	3	3	0	3
MDFC	1	1	2	2	0	2
PDFC	1	1	2	2	0	2
MANS	1	0	1	1	0	1
Metastatic DC	2	0	2	2	0	2
Invasive LC	8	0	8	7	1	8
Total	180	42	222	178	24	202

**Table 4:** Distribution of study subjects by PTEN and Her2/new immunohistochemical expression.

Lesion type	PTEN		Total	Her2/neu		Total
	−Ve	+ve		−Ve	+ve	
Carcinoma SBR	0	0	0	1	0	1
Infiltrating DC	3	30	33	39	8	47
Invasive DC	5	85	90	109	24	133
RIDC	0	2	2	1	1	2
Intracystic carcinoma	0	0	0	0	1	1
Ductal carcinoma in situ	1	10	11	3	0	3
MDF adenocarcinoma	0	11	11	2	0	2
PDFC	1	5	6	1	1	2
MANS	1	0	1	1	0	1
Metastatic ductal ca	0	5	5	2	0	2
Invasive lobular ca	0	4	4	6	2	8
Others	5	8	13	0	0	0
Total	**16**	**160**	**176**	165	37	202

**Table 5:** Distribution of age by P53 and BCL2 immunohistochemically expression.

Age	P53		Total	BCL2		Total
	−Ve	+ve		−Ve	+ve	
<25 years	0	6	6	1	0	1
26–35	22	9	31	26	2	28
36–45	56	10	66	60	10	70
46–55	44	8	52	50	4	54
56–65	20	4	24	20	6	26
66+	18	5	23	21	2	23
Total	160	42	202	178	24	202

## Data Availability

The data are available upon reasonable request to the author.
